# Pre-anesthetic assessment with three core questions for the detection of obstructive sleep apnea in childhood: An observational study

**DOI:** 10.1186/s12871-018-0483-y

**Published:** 2018-02-20

**Authors:** Joerg Schnoor, Thilo Busch, Nazar Turemuratov, Andreas Merkenschlager

**Affiliations:** 1 Department of Anesthesia and Intensive Care Medicine, Collm-Klinik-Oschatz, Parkstr. 1, 03435 Oschatz, Germany; 2 Department of Anesthesia and Intensive Care Medicine, University Hospital Leipzig, Liebigstr. 20, 04103 Leipzig, Germany; 3 Department of Neuropediatric, University Hospital Leipzig, Liebigstr. 20, 04103 Leipzig, Germany

**Keywords:** Obstructive sleep apnea, Sleep disordered breathing, Questionnaire, Pre-anesthetic assessment

## Abstract

**Background:**

Children with obstructive sleep apnea are at high risk for perioperative airway obstruction. Many “at risk” children may remain unrecognized. The aim of this study is to find a clinically practicable test to identify obstructive sleep apnea in childhood.

**Methods:**

In this pilot study, we prospectively compared four parental questionnaires with the respective findings of subsequent sleep laboratory testing in children. Right before sleep laboratory testing, children’s parents answered both the Pediatric Sleep Questionnaire, a subscale of the Sleep Related Breathing Disorder questionnaire (PSQ-SRBD-Subscale), and an eight-item questionnaire derived from it. Finally, we condensed the eight-item questionnaire to three core issues: Does your child regularly snore at night? Does your child demonstrate labored breathing during sleep? Does your child have breathing pauses during sleep? With it, two similar questionnaires were generated that differed in the formation of the resulting score. One questionnaire was built by a quotient comparable to the abovementioned questionnaires and a second as quick test that functioned as a simple sum score. Both sensitivity and specificity were determined by using a Receiver Operating Characteristic analysis.

**Results:**

In total, 53 children were included in the study. Both the PSQ-SRBD-questionnaire and self-derived eight-item questionnaire failed to reach statistically significant results in detecting obstructive sleep apnea. The set of three core questions with a score built by a quotient was statistically significant and provided sensitivity and a moderate specificity of 0.944 and 0.543, respectively. This could be slightly optimized by creating a simple sum-score (specificity of 0.571).

**Conclusions:**

The use of three core-questions may facilitate the detection of pediatric obstructive sleep apnea within the scope of the anesthesia survey. While the study has some limitations, future studies with both unselective collectives and older children might prove this ultra-short questionnaire to be advantageous in detecting pediatric OSA in clinical practices.

**Trial registration:**

German Clinical Trial Register (DRKS00010408, https://www.drks.de); date of registration 26.07.2016

## Background

Pediatric sleep-disordered breathing describes a continuum in upper airway obstruction that ranges from primary snoring to obstructive sleep apnea syndrome [1]. Children with obstructive sleep apnea (OSA) face a higher risk for perioperative airway obstruction and, unfortunately, many “at risk” children may remain unrecognized at the time of pre-anesthetic assessment [2]. While both the ASA and the STOP-BANG questionnaire demonstrate a reliable sensitivity and specificity in adults, those questionnaires are often of limited usefulness in childhood due to the complex nature of pediatric OSA [3–6]. For clinical practices, some tests have been developed in order to detect pediatric OSA [5, 7, 8]. Nevertheless, clinically practicable tests that identify pediatric OSA are still a challenge.

For daily clinically use, shortened questionnaires should provide both patient safety and save time. This appears to be of particular importance, since lack of time is a growing phenomenon for all health care providers. For this reason, a reliable “quick-test” on pediatric OSA is needed for a pre-anesthetic assessment in order to detect a hidden disease that may lead to serious respiratory sequels.

The aim of this pilot study was to create a questionnaire, which is as short as possible in order to match daily clinical life. Therefore, we prospectively compared four parental questionnaires with the respective findings of subsequent sleep laboratory testing in children.

## Methods

The local ethics committee approved this prospective observational trial (301-12-24092012, 18.09.12), which has been registered at German Clinical Trial Register (DRKS 00010408). All parents were informed about their voluntary and anonymous participation. Written informed consent was obtained before questionnaires were completed and on the day before the planned sleep laboratory examination was performed. All children who participated in the study were ordered to undergo a sleep laboratory for suspected OSA. Children were recruited as consecutive referrals. Exclusion criteria were over 18 years of age, anatomical airway obstructions, lack of capacity to consent, inadequate language skills, and refusal to participate.

### Questionnaires

At the time of admission, the children’s parents answered both a standard questionnaire (Pediatric Sleep Questionnaire subscale of the Sleep Related Breathing Disorder questionnaire, PSQ-SRBD-Subscale) and a self-derived OSA-short-questionnaire with eight typical questions (OSAsq8, Table 1) that were derived from the literature [6, 8]. Responses were “yes” = 1, “no” = 0, and “don’t know” = missing. The mean response on non-missing items is the score, which can vary from 0 to 1. The OSAsq8 includes OSA-specific questions about typical but secondary symptoms of an OSA that do not necessarily have to be present. In order to match clinical routines, the authors created, out of the OSAsq8, a further reduced set of three core questions (OSAsq3) that gives a score in the abovementioned manner. The three core questions focus on the pathognomonic and clinically visible symptoms of OSA: Snoring, labored breathing, and breathing pauses. The results of all the questionnaires PSQ-SRBD, OSAsq8, and OSAsq3 result as quotients from “yes” and “no” answers. In order to minimize possible failures in everyday clinical routines, the three core questions of the OSAsq3 were used to calculate an easy sum test (OSA3/8). Finally, the sensitivity and specificity of all tests were determined on the basis of a sleep laboratory examination.

**Table 1:** The short questionnaire for the detection of pediatric OSA (OSAsq8). Only the first three questions form the questionnaires OSAsq3 and OSA3/8, respectively

***1) Does your child regularly snore at night?***	
***2) Does your child demonstrate labored breathing during sleep?***	
***3) Does your child have breathing pauses during sleep?***	
4) Does your child have frequent infections?	
5) Does your child often demonstrate aggressive or hyperactive behavior?	
6) Does your child have a problem with daytime sleepiness?	
7) Is your child younger than 3 years?	
8) Has your child ever been treated for abnormalities in the oral and maxillofacial region?	

### PSQ-SRBD subscale

The PSQ-SRBD subscale consists of 22 closed response question-items. The scale has been validated against polysomnography and can be used for both research and clinical settings [8]. This test offered a sensitivity and specificity of 85% and 81%, respectively [9]. The mean response on non-missing items gives the score, which can vary from 0 to 1. It is suggested that a cut off value of 0.33 would be most effective in identifying pediatric OSA. The SRBD fits on one page. The survey needs about 3 min to take.

### OSA short questionnaire (OSAsq8)

The OSA-short questionnaire-8 (OSAsq8) consists of eight questions (Table 1). Like the PSQ-SRBD, the answers are either “yes”, “no” or “don’t know.” Analogous to the PSQ-SRBD subscale, the fraction of affirmative responses of non-missing items measures the score, which can vary from 0 to 1. The OSAsq8 fits on one page and takes about 1 min.

### OSAsq3 and OSA quick test (OSA3/8)

First, the OSA-short questionnaire 3 (OSAsq3) was subsequently condensed from the OSAsq8 and summarizes the first three key questions (Table 1): Does your child regularly snore at night? Does your child demonstrate labored breathing during sleep? Does your child have breathing pauses during sleep? Answers are either “yes”, “no,” or “don’t know.” Analogous to the abovementioned tests, the fraction of affirmative responses of non-missing items measures the OSAsq3, which can vary from 0 to 1. In the final step, the same three key questions were used to build a simple sum-score (OSA3/8). Here, answers with “yes” or “no” were rated with values of 2 (“yes”) and 1 (“no”), respectively. Unanswered questions were rated with zero. The sum of items gives ranges from 0 to 6. Finally, the OSA3/8 takes no more than 10 s.

### Sleep laboratory (OSAlab)

All children underwent sleep laboratory investigation (OSAlab) during the following night (University Hospital, Leipzig). The apnea hypopnea index (AHI) quantified obstructive events per hour. According to the literature, we defined childhood OSA as more than five obstructive events per hour [10]. While severity of pediatric OSA depends on various factors, i.e., total clinical pictures, duration of elevated end-tidal CO_2_, and frequency and severity of oxygen desaturation [6], one of the two experienced investigators assigned the children either to the group with OSA or without OSA, according to the local clinical standard.

### Statistical analysis

Continuous data are expressed as mean ± SD. Group comparison was conducted using both Mann-Whitney-U test and the Fisher’s exact test, where appropriated. A Receiver operating characteristic (ROC) analysis was used to investigate the relationship between each score value and the incidence of sleep apnea. ROC-curves were used in order to display the predictive value by plotting the true-positive rate (sensitivity) against the false-positive rate (1-specificity) at various threshold settings. The area under the curve (AUC) was compared to the area under the diagonal line of identity which corresponds to random chance (i.e. true positive rate equals false positive rate). In order to identify the corresponding numeric result for an optimal cut off point, the Youden-index (J) was calculated. This quantity is defined as J = maximum {sensitivity(x) – specificity (x) + 1} over all possible cut off points [11]. Statistical analysis was performed by using a computer-based program (SPSS, Version 20, IBM Corp. Armonk, NY). A two sided *P* value < 0.05 was defined statistical significance.

## Results

As part of this pilot study, 53 children were included in the study. There were 26 boys and 27 girls with an average age of 5.7 ± 4.3 years. Compared with the group of children without OSA (AHI 0,45 ± 0,9), we found less boys (*p* = 0,019) in the group of pediatric OSA (AHI 14 ± 13,4, Table 2).

**Table 2:** Biometrics and results (mean ± standard deviation). AHI = Apnea Hypopnea Index; PSQ-SRBD = Pediatric Sleep Questionnaire subscale of the Sleep Related Breathing Disorder questionnaire; OSAsq8 = OSA short questionnaire with eight items; OSAsq3 = OSA short questionnaire with three items; OSA3/8 = OSA questionnaire with three items as a sum-score. Data are expressed as mean ± SD. Group comparison was conducted using the Mann-Whitney-U test or the Fisher’s exact test*

	OSA (*n*=18)	Non-OSA (*n*=35)	*p*-value
AHI:	14 ± 13,4	0,45 ± 0,9	0,001
Male (n)	5	21	0,019*
Female (n)	13	14	0,019*
Age (years):	4,61 ± 2,5	6,31 ± 4,9	0,551
PSQ-SRBD:	0,50 ± 0,1	0,36 ± 0,2	0,013
OSAsq8:	0,53 ± 0,2	0,41 ± 0,2	0,060
OSAsq3	0,91 ± 0,3	0,59 ± 0,4	0,003
OSA3/8:	5,28 ± 1,0	3,57 ± 1,6	0,001

### OSAsq8 versus PSQ-SRBD subscale

**Fig. 1:**
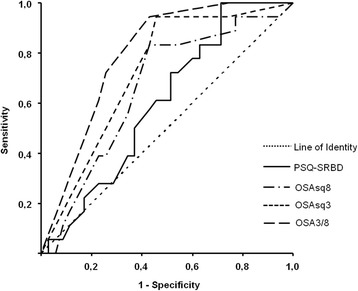
Resulting ROC curves of the Pediatric Sleep Questionnaire subscale of the Sleep Related Breathing Disorder questionnaire (PSQ-SRBD), OSA short questionnaires (OSAsq8, OSAsq3), and the quick-test OSA3/8. PSQ-SRBD, OSAsq8, and OSAsq3 depict the fraction of affirmative responses of non-missing items. The OSA3/8 functions as a simple sum-core

Confirmed by sleep laboratory testing (OSAlab), the short questionnaire that detects childhood-OSA (OSAsq8) reached a sensitivity and specificity of 0.833 and 0.571, respectively (cut-off 0.440; J-index 0.404). Thereby, specificity of the OSAsq8 was superior when compared to the PSQ-SRBD subscale (0.371), whereas sensitivity was found to be comparable (PSQ-SRBD: sensitivity 0,833; cut-off 0.340; J-index 0.204). In total, the predictive value of both OSAsq8 and PSQ-SRBD failed to reach statistical significance in detecting OSA in childhood (OSAsq8: *p* = .062, AOC 0.658; PSQ-SRBD: *p* = .240, AOC 0,599) when compared to random chance (Fig. 1).

### OSAsq3

OSAsq3 provided a sensitivity and specificity of 0.944 and 0.543, respectively. When compared to PSQ-SRBD, the OSAsq3 reached statistical significance in detecting OSA (*p* = .008; AOC 0.724; cut-off 0,584; J-index 0.487, Fig. 1).

### OSA3/8

Compared to the aforementioned tests, the OSA3/8-questionnaire provided a significant level in predicting pediatric OSA (*p* < .001) and reached a high sensitivity (0.944) with a specificity of 0.571 (AOC 0.794; cut-off 3.5; J-index 0.515, Fig. 1).

## Discussion

Today, there are several pediatric OSA screening questionnaires. Although some of them provide good sensitivity and specificity, the complexity of completion might limit these tests for clinical use [7]. Thus, simple and less time-consuming questionnaires are of particular importance in screening children for having OSA. We found that the OSA3/8 is a simple sum-test that will take only few seconds.

In line with childhood obesity, the incidence of pediatric OSA is expected to further increase [12]. The validated PSQ-SRBD is based on 22 question items, out of which the score has to be calculated [8]. Thus, the PSQ-SRBD survey needs several minutes and can become inadvertently inaccurate. Moreover, as our results suggest, the PSQ-SRBD failed to predict OSA, as we used the given cut-off. This result is quite surprising, and we might speculate on this, but the validation of the PSQ-SRBD was not the focus of this study. Instead, the results further suggest that the OSA3/8 questionnaire shows a clear advantage in predicting childhood OSA with respect to sensitivity, specificity, and time expenditure.

Gasparini and colleagues [13] already described a simplified test for OSA in children: the obstructive airway child test (OACT). This test focuses on clinical signs and symptoms. This test works by using 12 typical questions originally validated for adults. The authors concluded that this questionnaire offers an efficient method to evaluate and diagnose childhood OSA.

Kadmon and colleagues [7] created an eight-item questionnaire (IF SLEEPY) with high sensitivity and low specificity for OSA diagnosis in primary care settings. Beyond snoring, labored breathing, and breathing stops, this test focuses on anatomical, biometric, and psychodynamic parameters, which do not have to occur in all children.

Raman et al. [5] identified questions related to OSA that were adapted from the SRBD. Similar to our study, they attempted to reduce the set of questions in order to obtain a more practical predictive tool. Their shortened test works on a six-question scale. On one hand, their test seems more practicable and time efficient. On the other hand, the test focuses on older children between the ages of six and 18 years. This test might be an advantage for children whose parents seldom witness them.

For young children, Tait and colleagues [14] offer a simplified and shortened five-item questionnaire (STBUR) that focuses on main symptoms like snoring, difficulty breathing, and daytime sleepiness. While both snoring and difficult breathing demonstrate symptoms and causes of OSA at the same time, daytime sleepiness is a resulting note, which can be the consequence of various causes and, therefore, might be less specific in detecting pediatric OSA.

When compared to the abovementioned tests, the OSA3/8 promises both good sensitivity and specificity. This quick-test focuses on three key symptoms of nocturnal airway obstructions. With it, the OSA3/8 works as a simple functional sum-scale that will take only few seconds. Its three key questions focus on breathing patterns and are presented as follows:

1. Does your child regularly snore at night?
2. Does your child demonstrate labored breathing during sleep?
3. Does your child have breathing pauses during sleep?

If, at least, two out of the three issues are confirmed with “yes” (≥ 4 points), the child could be suspected of having OSA. With this result, either the child should be supplied for further diagnostics, or post-anesthesia monitoring should be adapted accordingly. Among our childs with OSA only one had an OSA3/8 score below the cut off 3.5. In this study, only two cases reached a sum of of 2 points by only acknowledging snoring, while the parents excluded both labored breathing and breathing pauses. This main clinical disadvantage should guide clinicians to search for further indications in order to differentiate between a simple snorer and a child at risk for having OSA. If in doubt, it would be safer to consider this child as a patient with OSA.

This practical approach makes the OSA3/8 a quick test that promises patient safety and time efficiency during the pre-anesthetic assessment. The short sum-scale may reduce the risk of errors when compared to scales that are based on both multiple items and the formation of a quotient.

This study shows some limitations. First, we tested the OSA3/8 in children who were already suspicious they had OSA and who were already planning to be diagnosed in a sleep laboratory. In order to find the true sensitivity and specificity of the three core questions as a screening tool, our findings must be validated in children who are not already suspected to have OSA. Second, due to a residual blur in diagnosis and classification of an OSA, the diagnosis was made by one investigator. While this procedure should reduce systematic errors in diagnosis, last doubts cannot be eliminated. Third, the OSA3/8 score was determined in children between the age of two and 16 years (mean age 6 years) and it is based solely on parental observations. However, our investigation focused on younger children who may more frequently experience the nightly care of their parents. Thus, the older the children are, the more difficult the test may be, if children might less frequently be observed while sleeping. Fourth, the OSA3/8 does not account for biometric data. For example, we did not focus on the BMI. However, their values might be questionable, since the body mass index (BMI) and neck circumference failed to improve test significance [5, 15]. Over all, the controversial findings in the literature might be due to the patients’ age itself. OSA in younger children is mainly associated with tonsillar hypertrophy. In contrast, older children and adolescents more frequently present obesity as a symptom. With growing age and obesity, biometrics might gain importance, comparable with adulthood. Except for craniofacial disorders, this might indicate that we can neglect biometric abnormities for predicting OSA in younger children. Fifth, we did not use the opportunity to retest our questionnaire. Similar to the work by Raman and colleagues, the set of questions that forms the OSA3/8 was constructed from the previously validated PSQ-SRBD and, conclusively, it is suggested that a test retest validation might be not necessary [5].

## Conclusions

In total, the OSA3/8 might offer an easily applied tool to detect pediatric OSA for pre-anesthetic assessments. Despite all the limitations of this study, the three core questions might prove itself in clinical routines better than extensive questionnaires that do not seem to always be helpful in daily clinical life. The OSA3/8 might be a promising approach in delivering appropriate diagnoses and care to these patients.

## Abbreviations

AHI: Apnea Hypopnea Index
AUC: Area Under the Curve
OACT: Obstructive Airway Child Test
OSA: Obstructive Sleep Apnea
OSA3/8: Three OSA-core-questions out of eight questions
OSAlab: Sleep laboratory for detecting OSA
OSAsq3: OSA-Short-questionnaire-3 questions
OSAsq8: OSA-Short-questionnaire-8 questions
PSQ: Pediatric Sleep Questionnaire
ROC: Receiver Operating Characteristic
SRBD: Sleep Related Breathing Disorder
STBUR: Snoring, Troubled Breathing, and Un-Refreshed questionnaire
